# In Vitro Characterization of Hypoxia Preconditioned Serum (HPS)—Fibrin Hydrogels: Basis for an Injectable Biomimetic Tissue Regeneration Therapy

**DOI:** 10.3390/jfb10020022

**Published:** 2019-05-13

**Authors:** Ektoras Hadjipanayi, Philipp Moog, Sanjar Bekeran, Katharina Kirchhoff, Andrei Berezhnoi, Juan Aguirre, Anna-Theresa Bauer, Haydar Kükrek, Daniel Schmauss, Ursula Hopfner, Sarah Isenburg, Vasilis Ntziachristos, Milomir Ninkovic, Hans-Günther Machens, Arndt F. Schilling, Ulf Dornseifer

**Affiliations:** 1 Experimental Plastic Surgery, Department for Plastic and Hand Surgery, Klinikum rechts der Isar, Technische Universität München (TUM), D-81675 Munich, Germany; e.hadjipanayi@gmail.com (E.H.); philippmoog@web.de (P.M.); sanjar.bekeran@gmx.de (S.B.); katharina.kirchhoff@icloud.com (K.K.); anna.theresa.bauer@googlemail.com (A.-T.B.); haydarkuekrek@gmx.de (H.K.); schmauss.daniel@gmail.com (D.S.); ursula.hopfner@tum.de (U.H.); dornseifer@ustransplant.de (U.D.); 2 Department of Plastic, Reconstructive and Aesthetic Surgery, Isar Klinikum, 80331 Munich, Germany; 3 Department of Plastic, Reconstructive, Hand and Burn Surgery, Bogenhausen Hospital, 81925 Munich, Germany; dr.isenburg@neuhannlorenz-isenburg.com (S.I.); ninkovic@me.com (M.N.); 4 Institute of Biological and Medical Imaging, Helmholtz Zentrum Munich; Deutsches Forschungszentrum für Gesundheit und Umwelt (GmbH), 85764 Neuherberg, Germany; andrei.berezhnoi@helmholtz-muenchen.de (A.B.); Juanaguir@gmail.com (J.A.); v.ntziachristos@tum.de (V.N.); 5 Department for Plastic, Reconstructive and Aesthetic Surgery, Ospedale Regionale di Lugano, 6900 Lugano, Switzerland; 6 Department of Trauma Surgery, Orthopedics and Plastic Surgery, Universitätsmedizin Göttingen, D-37075 Göttingen, Germany; arndt.schilling@med.uni-goettingen.de

**Keywords:** hypoxia, angiogenesis, growth factor, injectable hydrogel, fibrin matrix, peripheral blood cells, tissue regeneration

## Abstract

Blood-derived growth factor preparations have long been employed to improve perfusion and aid tissue repair. Among these, platelet-rich plasma (PRP)-based therapies have seen the widest application, albeit with mixed clinical results to date. Hypoxia-preconditioned blood products present an alternative to PRP, by comprising the complete wound healing factor-cascade, i.e., hypoxia-induced peripheral blood cell signaling, in addition to platelet-derived factors. This study set out to characterize the preparation of hypoxia preconditioned serum (HPS), and assess the utility of HPS–fibrin hydrogels as vehicles for controlled factor delivery. Our findings demonstrate the positive influence of hypoxic incubation on HPS angiogenic potential, and the individual variability of HPS angiogenic factor concentration. HPS–fibrin hydrogels can rapidly retain HPS factor proteins and gradually release them over time, while both functions appear to depend on the fibrin matrix mass. This offers a means of controlling factor retention/release, through adjustment of HPS fibrinogen concentration, thus allowing modulation of cellular angiogenic responses in a growth factor dose-dependent manner. This study provides the first evidence that HPS–fibrin hydrogels could constitute a new generation of autologous/bioactive injectable compositions that provide biochemical and biomaterial signals analogous to those mediating physiological wound healing. This therefore establishes a rational foundation for their application towards biomimetic tissue regeneration.

## 1. Introduction

A wound is characterized as a disorder of tissue structure and function [1,2] that, under physiological conditions, is regenerated through a series of defined wound healing stages: haemostasis, inflammation, proliferation, and angiogenesis, and eventually tissue remodeling [2,3]. Haemostasis and angiogenesis are two closely interlinked processes, that upon vascular injury operate in harmony, i.e., sequentially, to re-establish the microcirculation to its former state [2], thus supporting tissue repair. It has previously been demonstrated that this precise transition from coagulation to granulation tissue formation may be both biochemically and biomechanically regulated through the fibrin matrix, via its role as protein factor carrier [4], as well as cell scaffold [5,6].

Of the many biological forces that regulate new vessel formation within regenerating tissue, hypoxia is the strongest stimulus for angiogenesis [7,8,9]. Utilization of hypoxia as a tool for angiogenic induction harnesses the innate biological mechanism(s) that naturally generates angiogenesis in the body, in physiological states e.g., embryogenesis, as well as pathological conditions e.g., wound healing, ischemia, tumor formation [7,10,11]. We previously developed the first concepts for therapeutic angiogenesis based on this idea, using dermal fibroblasts, which were employed as growth factor producing cells, integrated into implantable/injectable systems [12,13,14]. This provided a platform for the controlled release of hypoxia-induced protein factors, which has been shown in vivo to be effective in promoting vascularization, oxygenation, and integration of subcutaneously implanted collagen matrices [12,13]. As a further development of this approach we have tested peripheral blood cells (PBCs) as growth factor providers [15]. PBCs respond to hypoxia by upregulating angiogenic factor signaling (e.g., VEGF [15,16,17,18], bFGF [17,18], IL-8 [18], MMP-9 [18]). Indeed, among the various candidate cell types that are suitable for hypoxic stress stimulation, PBCs are ideal, due to their easy harvest and ample availability [15]. Moreover, autologous cells provide the advantage of a lower risk for adverse immune reactions and infection, compared to allogeneic or xenogeneic cells [19,20]. Thus, the provision of a standardized ex vivo hypoxic microenvironment for PBCs, which simulates that of an in vivo wound, could aid the obtainment of physiological pro-angiogenic growth factor mixtures, at naturally occurring concentrations and ratios [15]. This admittedly offers an improved alternative to simply concentrating platelet-derived factors to supra-physiological levels, e.g., platelet-rich plasma (PRP), an approach that currently represents the gold-standard of blood-based regenerative therapies.

**Figure 1:**
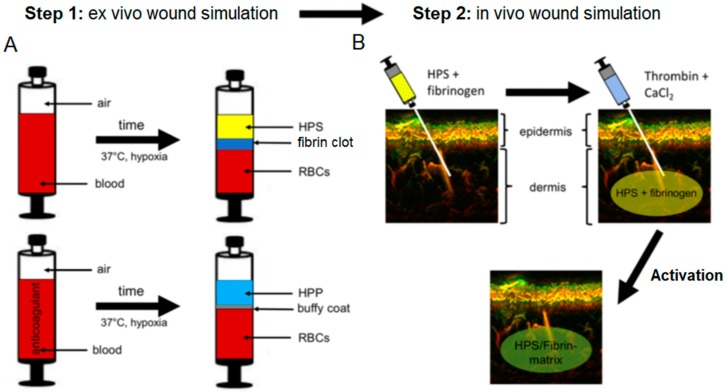
Novel clinical approach for injectable tissue therapy using the extracorporeal wound simulation method. (**A**) Step 1: Blood is allowed to clot, or anticoagulated, and peripheral blood cells (PBCs) in the fibrin clot or buffy coat, respectively, are preconditioned under pericellular (local) hypoxia (~1% O_2_) and physiological temperature (37 °C) for 4 to 8 days. Sedimentation passively separates growth factor-rich hypoxia preconditioned serum (HPS) or hypoxia preconditioned plasma (HPP) from red blood cells (RBCs). (**B**) Step 2: Due to the consumption of fibrinogen for clotting during blood incubation, fibrinogen must be replaced by adding it to HPS. After injection of the HPS/fibrinogen solution into the dermis, thrombin/CaCl_2_ is injected at the same site for activation and formation of a HPS–fibrin hydrogel matrix.

Hypoxia preconditioned blood products, generated through the process of ‘extracorporeal wound simulation’, i.e., peripheral blood incubation under physiological hypoxia and temperature, could provide optimized autologous secretomes that can support angiogenesis and promote tissue repair on-demand, since the factor composition is defined by the physiological and patient-specific cellular responses that mediate normal wound healing [21,22]. These blood-derived growth factors can be delivered in the form of serum, i.e., hypoxia preconditioned serum (HPS) or plasma, i.e., hypoxia preconditioned plasma (HPP), by adjusting blood coagulation (Figure 1A, note; HPS contains a greater amount of platelet-derived growth factors than HPP, as a result of coagulation-mediated platelet activation). When administering such secretomes in vivo, controlled growth factor delivery might be achieved by activating HPP and HPS with thrombin/calcium, which rapidly leads to the formation of a fibrin gel-matrix at the injection site, that then serves as protein factor carrier [21,23,24]. Since fibrinogen in HPS is consumed for clotting during blood incubation, it must be replenished through the addition of exogenous fibrinogen (Figure 1B). Despite this limitation, HPS can be viewed as offering a more ‘complete’, and by extension, physiological secretome compared to its HPP counterpart, since it comprises both the coagulation-mediated phase, i.e., platelet-derived growth factors, and the hypoxia-induced signaling phase, i.e., growth factors produced by PBCs in response to hypoxic exposure, of the wound healing cascade [22].

**Figure 2:**
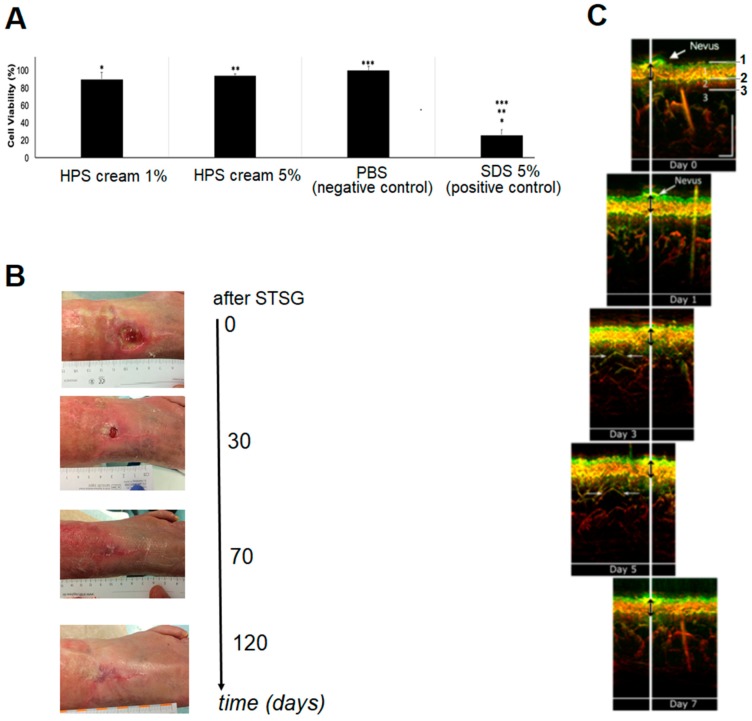
Preliminary assessment of HPS in vivo biocompatibility. (**A**) Plot showing cell viability results obtained through EpiSkin test of a 1% and 5% HPS-containing cream product. Phosphate buffered saline (PBS) and Sodium Dodecyl Sulphate (SDS) 5% aq. solution were tested as negative and positive controls, respectively. *, **, *** = *p* < 0.01 (*n* = 3). Error bars represent s.d. (**B**) Example of treatment of a chronic venous leg (pretibial) ulcer (~3 cm diameter) in a 63-year-old female patient through HPS topical administration (5% HPS-containing ointment), within 3 months. (**C**) Example of subject receiving a forearm injection of HPS–fibrin hydrogel, where the hydrogel-induced local tissue distortion was analyzed through an UB-RSOM system for 7 days following injection. Numbered compartments in Day 0 indicate the epidermal layer (1), capillary loops at the epidermal–dermal junction (2), and upper plexus of the dermal layer (3). The white vertical line indicates position of a nevus, in close proximity to the injection site, captured at different days. Vertical bidirectional black arrows, overlaid with the vertical white line, indicate the measured average width from the centre of the nevus to the epidermal–dermal junction. Opposing horizontal white arrows indicate a vascular structure captured at Day 3 and Day 5.

HPS has already been employed clinically, by our group, as a bioactive topical therapy to aid wound regeneration in selected cases, e.g., in leg and foot ulcers persisting after gold-standard treatment. Despite the promising results available to date (see case report in results, Figure 2B), growth factor penetration into the dermis evidently remains a limitation of topical application. Consequently, the ability to deliver HPS through injectable administration could further enhance the secretome’s bioavailability within the wound bed, thus providing the regenerative signals within healthy tissue layers, from which repair can be initiated. 

In this study, we set out to extend our knowledge about the behavior of HPS–fibrin hydrogels, as vehicles for controlled delivery of HPS growth factors. We performed a range of in vitro experiments to assess pro- and anti-angiogenic protein factor loading and release kinetics, and how these relate to the mass and volume of the fibrin hydrogel matrix, parameters that, as we show, can be precisely controlled by adjusting the HPS fibrinogen concentration. Our findings, supported by an in vitro analysis of HPS factor concentration-dependent induction of cellular angiogenic responses, point to the suitability of HPS–fibrin hydrogels as growth factor carriers and bioactive scaffolds for tissue repair and regeneration.

## 2. Results

### 2.1. Preliminary Assessment of HPS In Vivo Biocompatibility

Our clinical experience with HPS to date indicates that it is safe to use for autologous administration. As a first step towards characterization of the safety profile of this secretome, we tested the ability of HPS to be an irritant to skin, when it is applied topically. A custom-made cream product containing 1% or 5% HPS was subjected to an in vitro skin irritation test using the EPISKIN model (according to the OECD Test Guideline No. 439, 26 July 2013), which analyzes the irritation potential of a substance by measurement of its cytotoxic effect, as reflected in the MTT assay. Neither 1% nor 5% HPS concentration products showed significantly reduced cell viability in comparison to phosphate buffered saline (PBS), used here as negative control (mean value 90%), with all obtained viability results being above 50% (Figure 2A). These findings indicated that both HPS concentrations tested could be considered to be non-irritant to skin. In vivo topical administration of HPS in chronic wound ulcers has also proven to be safe, and also effective in aiding full wound closure and epithelialization within three months from starting treatment, in patients where gold-standard surgical treatment with repeated wound debridement and split-thickness skin grafting (STSG) had previously failed to heal the wound (case report, Figure 2B).

The in vivo biocompatibility of HPS was further characterized by assessing the safety of dermal injectable application of HPS–fibrin hydrogels. The local tissue distortion induced by the HPS–fibrin hydrogel was analyzed for seven days following intradermal injection using an UB-RSOM system (Figure 2C). The measured mean epidermal thickness was 125.4 ± 12.8 µm (Day 0–Day 7). To evaluate microvasculature distortion in the superficial dermis, we analyzed the same vascular structure, captured at Day 3 and Day 5. The measured average structure length was 828 µm and 840 µm for Day 3 and Day 5, respectively. The consistency of the obtained epidermal thickness results and vessel position analysis indicated that HPS injection allows uniform interstitial integration of the fibrin gel-matrix, without noticeable distortion of local tissue architecture, i.e., epidermal–dermal topography. Importantly, no side effects or allergies were observed during or after treatment. 

### 2.2. Proteomic Analysis of Releasates from Fibrin Matrices Cultured under Wound-Simulating Conditions

**Figure 3:**
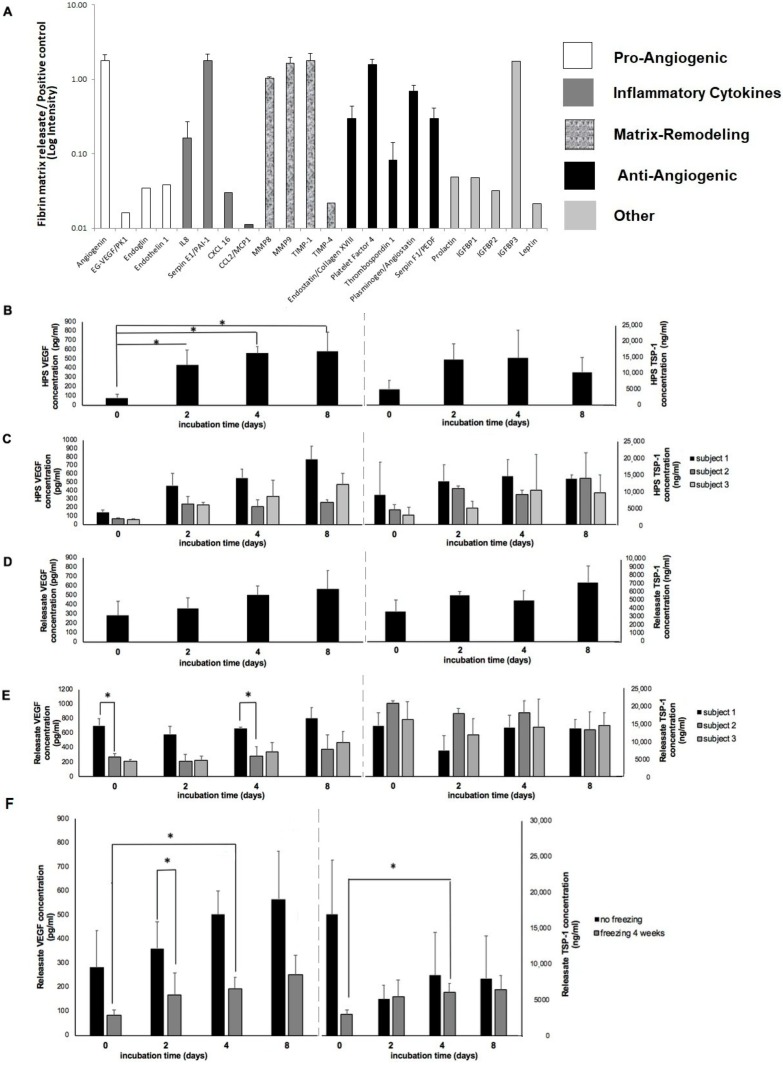
Proteomic analysis of fibrin matrix releasates and the effect of varying blood incubation time on HPS and HPS–fibrin hydrogel releasate VEGF/TSP-1 concentration. (**A**) Plot showing the proteomic profile of factor proteins detected in releasates of fibrin hydrogels that were incubated for 7 days in the presence of PBCs cultured under hypoxia (3% O2) (*n* = 3). (**B**) Plot showing HPS VEGF and TSP-1 concentrations for blood incubation times of 0, 2, 4, and 8 days, * = *p* < 0.01 (*n* = 5). (**C**) Plot showing individual subject analysis of HPS VEGF and TSP-1 concentrations for the previous conditions (*n* = 3). (**D**) Plot showing HPS–fibrin hydrogel releasate VEGF and TSP-1 concentrations for blood incubation times of 0, 2, 4, and 8 days (*n* = 5). (**E**) Plot showing individual subject analysis of HPS–fibrin hydrogel releasate VEGF and TSP-1 concentrations for the previous conditions, * = *p* < 0.05 (*n* = 3). (**F**) Plot comparing the VEGF and TSP-1 concentrations in releasates of HPS–fibrin hydrogels immediately after the preconditioning period (0, 2, 4, and 8 days) and following storage for 4 weeks at −20 °C degrees, * = *p* < 0.05 (*n* = 5). Error bars represent s.d.

The first step towards assessing the utility of fibrin matrices as potential carriers of hypoxia preconditioned blood-derived secretomes was to carry out a proteomic analysis of releasates obtained from fibrin hydrogels that were cultured in the presence of PBCs under hypoxia (3% O_2_). A previously described 3D culture model that simulates the wound microenvironment (hemostatic components model, HCM) [4]) was used to ensure no direct contact between the fibrin matrix and PBCs during the culture period, in order to prevent any passive factor loading/direct cell factor release into the matrix. Analysis of fibrin matrix releasates, obtained through matrix centrifugation after 7 days of culture, revealed that a range of protein factors were present, including pro- and anti-angiogenic factors, matrix-remodeling proteins and inflammatory cytokines (Figure 3A), indicating active binding and/or trapping of these factors by the fibrin matrix. 

### 2.3. Effect of Varying the Length of Hypoxic Preconditioning on HPS and HPS–Fibrin Hydrogel Releasate VEGF/TSP-1 Concentration

The length of hypoxic blood incubation was the primary environmental factor investigated as a means of assessing pro- and anti-angiogenic factor production by PBCs, as reflected by HPS factor levels. Physiological hypoxia (~1% O_2_) was generated here in situ through cell-mediated O_2_ consumption, under the specified blood volume per unit area of the incubation chamber (BVUA ≥ 1 mL/cm^2^, see methods Section 4.4) [4,21]. The HPS concentration of the pro-angiogenic factor VEGF was found to be significantly higher after 2, 4, and 8 days of incubation as compared to fresh serum (no incubation) (*p* < 0.01) (Figure 3B). In contrast, the HPS concentration of the angiogenic inhibitor TSP-1 followed a bell-shape pattern, peaking at 4 days and falling towards baseline by 8 days of incubation, although differences were not statistically significant. No significant differences in VEGF or TSP-1 levels were observed between the three incubation periods tested, an effect persisting when individual subject analysis was carried out (Figure 3C).

We then analyzed the concentration of these factors in HPS–fibrin hydrogel releasates, obtained through hydrogel centrifugation in fresh medium. According to our previous results, VEGF cumulative releasate concentration showed a rising trend with increasing blood incubation length (Figure 3D), although the increase observed was non-significant this time around, likely due to a higher serum baseline level. Significant differences in VEGF releasate levels were, however, found between subjects at 0 and 4 days of incubation (*p* < 0.05) (Figure 3E), in alignment with the previously observed, yet non-significant inter-subject differences in serum and HPS VEFG levels (Figure 3C). TSP-1 cumulative releasate concentration appeared mostly unchanged over the tested incubation period range (Figure 3D,E).

To identify whether hypoxia preconditioned products can be stored at low temperature, which would be a clinically useful property for off-the shelf application, the releasates of HPS–fibrin hydrogels (obtained through hydrogel centrifugation in fresh medium) were frozen for 4 weeks at −20 °C, and then factor bioavailability was tested via ELISA. As shown in Figure 3F, VEGF concentration in frozen releasates showed a drop compared to fresh samples, although this decrease was only significant for the 2 day blood incubation period (*p* < 0.05). Nonetheless, VEGF remained always detectable in frozen samples, and importantly, maintained a significantly higher level in 4-day blood incubation samples compared to freeze-stored basal serum-derived hydrogel releasates (no blood incubation) (*p* < 0.05) (Figure 3F). In contrast, TSP-1 releasate concentration remained largely unchanged following freezing, but did also exhibit a significant increase from 0 to 4 days of blood incubation in frozen samples (*p* < 0.05) (Figure 3F).

### 2.4. Analysis of HPS-Induced Endothelial Cell and Fibroblast Responses

**Figure 4:**
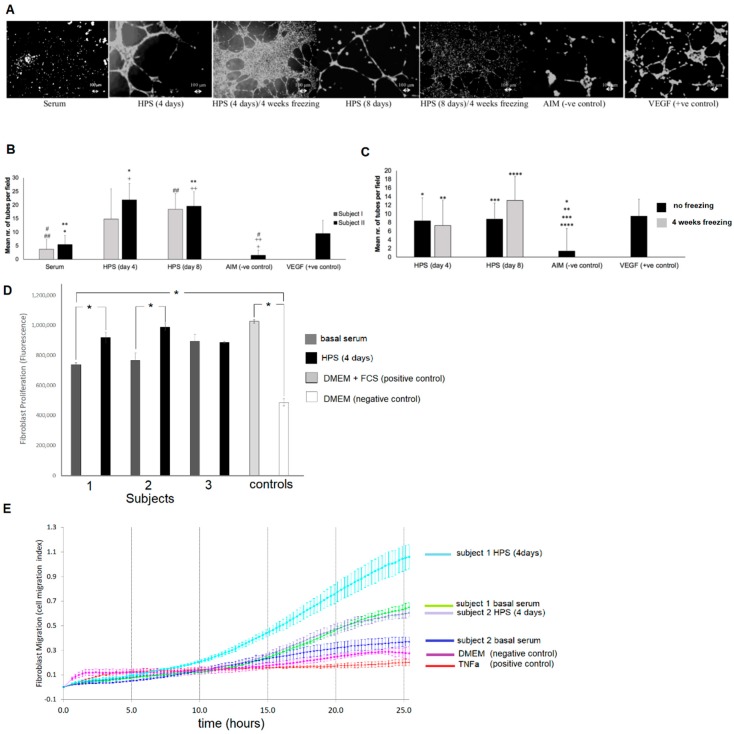
Effect of HPS on endothelial cell angiogenic responses and dermal fibroblast proliferation and migration in vitro. (**A**) Image panel showing the angiogenic response of human umbilical vein endothelial cells, cultured for 12 h under serum, fresh HPS and HPS frozen for 4 weeks at −20 °C (4 and 8 days of blood incubation), serum-free AIM medium (negative control) and VEGF (positive control). (**B**) Plot comparing the induced tube formation for serum and HPS (4 and 8 days of blood incubation), with secretomes derived from 2 subjects. *, **, #, ##, +, ++ = *p* < 0.01 (*n* = 3). (**C**) Plot comparing the induced tube formation for fresh HPS and HPS frozen for 4 weeks at −20 °C (4 and 8 days of blood incubation). *, **, ***, **** = *p* < 0.01 (*n* = 3). (**D**) Plot comparing the proliferation of human dermal fibroblasts, cultured in medium (DMEM) containing 10% HPS (4 days of blood incubation) or 10% basal serum for 72 h. DMEM without or with 10% fetal calf serum (FCS) was tested as negative and positive control, respectively. * = *p* < 0.05 (*n* = 5). (**E**) Plot showing the migration of human dermal fibroblasts, over 26 h, toward chemoattractant medium; DMEM containing 10% HPS (4 days of blood incubation) or 10% basal serum. DMEM and TNFα were tested as negative and positive controls, respectively. Error bars represent s.d.

Our current clinical results indicate that HPS accelerates closure of chronic ulcers when applied topically to the wound (Figure 2B). In an effort to understand the underlying cellular mechanism(s) supporting this process, we first examined the angiogenic effect of hypoxia preconditioned serum on human umbilical vein endothelial cells. HPS, derived after both 4 and 8 days of blood incubation, was found to be significantly more potent than serum in inducing tube formation in vitro (*p* < 0.01) (Figure 4A,B). While its pro-angiogenic effect persisted following 4 weeks of freeze storage at −20 °C (Figure 4A,C), in accordance to the VEGF data of Figure 3F, more extensive endothelial cell clustering was observed with these secretomes (Figure 4A).

Furthermore, we tested the hypothesis that HPS exerts its regenerative effect by also influencing stromal cell behavior. The proliferation of human dermal fibroblasts was assessed using the alamar blue assay, which showed that HPS was more potent than basal serum in stimulating fibroblast proliferation, in two out of the three subjects tested (*p* < 0.05) (Figure 4D). However, no significant difference was found between HPS and fetal calf serum (FCS), tested here as positive control. Similarly, HPS induced fibroblast directional migration through a microporous membrane more strongly than basal serum, an effect that first became apparent 10 h after the start of the experiment and was maintained at 26 h (Figure 4E). Additionally, the level of fibroblast migration was found to vary between subjects, while this difference was observed in both serum and HPS cultures (Figure 4E).

### 2.5. Effect of Varying HPS Fibrinogen Concentration on HPS–Fibrin Hydrogel Mass and Volume

**Figure 5:**
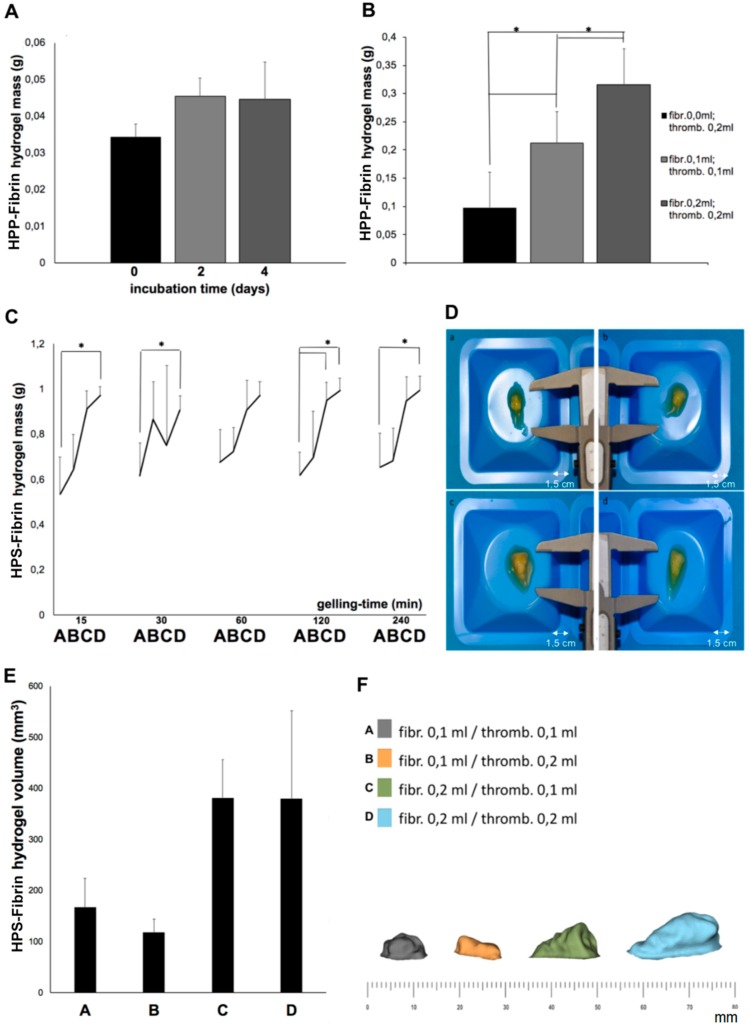
Effect of varying the HPP/HPS fibrinogen concentration on HPP–/HPS–fibrin hydrogel mass, and 3D-scan volumetric analysis of HPS–fibrin hydrogels. (**A**) Plot showing the effect of varying blood incubation time (0, 2, 4 days) on HPP–fibrin hydrogel mass without addition of exogenous fibrinogen (*n* = 5). (**B**) Plot showing the effect of varying HPP fibrinogen/thrombin concentration on HPP–fibrin hydrogel mass (HPP incubation 4 days), * = *p* < 0.001 (*n* = 5). (**C**) Plot showing the influence of varying gelling time (15 to 240 min) and HPS fibrinogen/thrombin concentration on HPS–fibrin hydrogel mass (HPS incubation 4 days). A = fibrinogen 0.1 mL, thrombin 0.1 mL; B = fibrinogen 0.1 mL, thrombin 0.2 mL; C = fibrinogen 0.2 mL, thrombin 0.1 mL; D = fibrinogen 0.2 mL, thrombin 0.2 mL, * = *p* < 0.05 (*n* = 5). (**D**) Photographs of HPS–fibrin hydrogels for conditions A, B, C, D same as above (bars = 1.5 cm). (**E**) Plot showing the effect of varying HPS fibrinogen/thrombin concentration (conditions A, B, C, D same as above) on HPS–fibrin hydrogel volume, detected via 3D scan (*n* = 4). (**F**) 3D scan-generated virtual models of HPS–fibrin hydrogels for the four fibrinogen/thrombin concentration conditions tested. For D, E, and F: HPS incubation 4 days, gelling time = 4 h. Error bars represent s.d.

The key hypothesis put to the test in this study was that fibrin hydrogels could provide suitable carriers for HPS protein factors. The ability to control the carrier size, i.e., its mass and volume, would logically determine the ability to adjust the carrier’s growth factor capacity, and therefore the degree of releasate factor dose control. To establish a baseline, we first examined the effect of varying the blood incubation time and plasma fibrinogen concentration on HPP–fibrin hydrogel mass. As shown in Figure 5A, the length of blood incubation (2 vs. 4 days) did not significantly influence the mass of HPP–fibrin hydrogels, obtained following thrombin activation, when no extra fibrinogen was added to HPP. In contrast, hydrogel mass correlated significantly with the volume of exogenous plasma fibrinogen added to HPP (*p* < 0.001) (Figure 5B). Based on these findings, the influence of varying the gelling time (following thrombin activation) and HPS fibrinogen concentration (9 vs. 18 mg/mL) on the mass of HPS–fibrin hydrogels were then examined. The results in Figure 5C show that while the mass of HPS–fibrin hydrogels was more or less independent of gelling time (15–240 min), a significant (*p* < 0.05) and almost proportional increase in hydrogel mass and size (Figure 5D), could be achieved by doubling the volume of added fibrinogen, in accordance with the HPP results. Importantly, the increase in hydrogel mass was always greater than what was expected from the volume of fibrinogen solution added to HPS (Figure 5C, note: the shape of the mass curves obtained suggests that thrombin solution volume acted additively to total gel volume), indicating efficient absorption of HPS into the formed fibrin hydrogel matrix, i.e., ≥50% of initial HPS wet volume (≥0.5 mL), assuming that the full volume of fibrinogen/thrombin solution was integrated into the gel.

In order to identify whether varying the HPS fibrinogen concentration also correlated with changes in hydrogel volume, i.e., the three-dimensional stability of hydrogels, 3D scan-assisted quantification of hydrogel volume differences was carried out. In agreement with the mass measurements, the volume of hydrogels was found to increase by two-fold after doubling the HPS fibrinogen concentration (9 vs. 18 mg/mL), an effect not seen when only the thrombin concentration was increased (Figure 5E). However, actual hydrogel volumes were somewhat lower compared to what was expected from the mass measurements above, suggesting some degree of fluid loss during the scanning process. The proportional increase in volume observed with this method, although statistically not significant, was also visible macroscopically in the 3D scan-generated virtual models of hydrogels (Figure 5F). 

### 2.6. Assessment of the Factor Retention Capacity of HPS–Fibrin Hydrogels and the Effect of Varying Hydrogel Mass on Factor Retention Rate and Factor Release Rate

**Figure 6:**
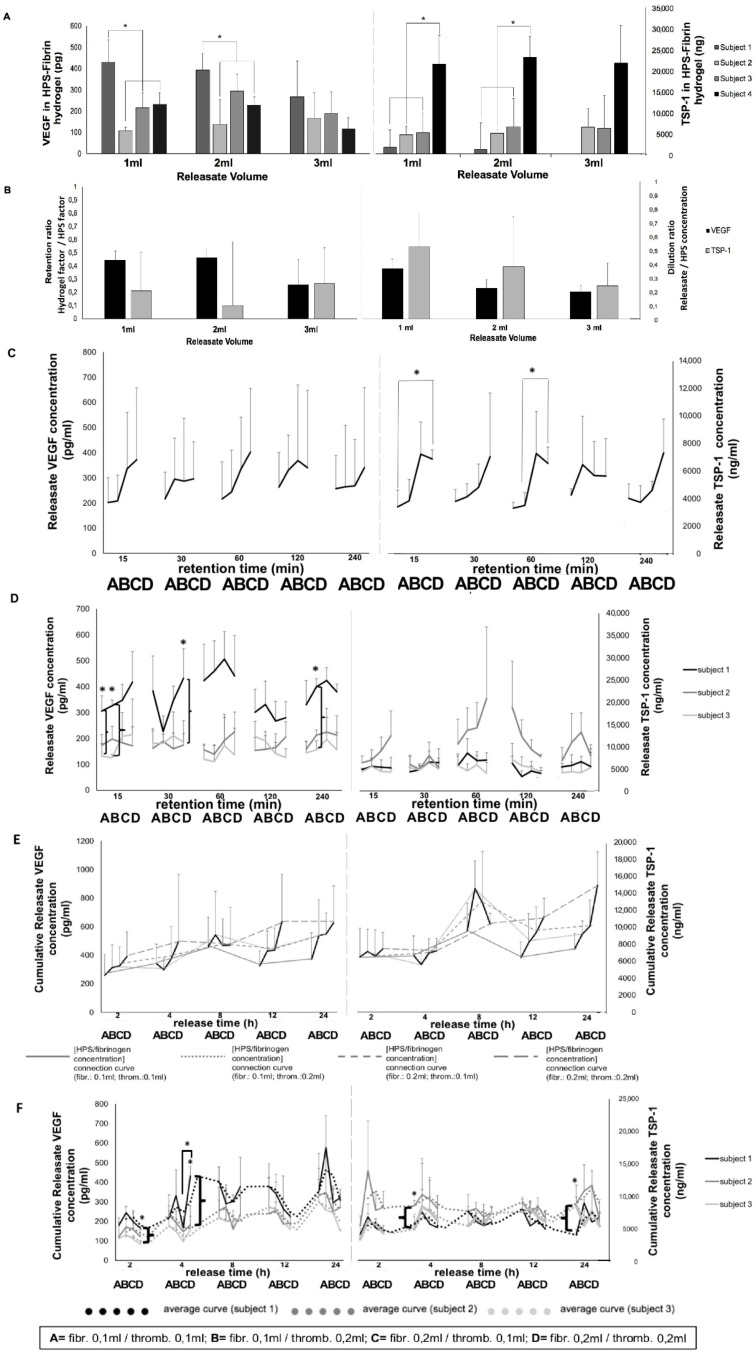
Analysis of factor retention capacity of HPS–fibrin hydrogels and the effect of varying HPS–fibrin hydrogel mass on factor retention and release rate. (**A**) Plot showing the calculated amount of VEGF and TPS-1 retained within HPS–fibrin hydrogels, after 24 h release, for individual subjects and different releasate volumes (1–3 mL), * *p* < 0.05 (*n* = 4). (**B**) Plot showing the fraction of HPS VEGF and TPS-1 retained within HPS–fibrin hydrogels (retention ratio) following 24 h release, and the dilution ratio of releasate/HPS factor concentration (*n* = 4). (**C**) Plot showing VEGF and TSP-1 releasate concentration under a variable HPS fibrinogen concentration (A, B, C, D) and retention time (15–240 min), * = *p* < 0.05 (*n* = 5). (**D**) Plot showing individual subject analysis for the above conditions, * = *p* < 0.05 subject 1 vs. 3 (vertical brackets) (*n* = 3). (**E**) Plot showing VEGF and TSP-1 cumulative releasate concentration under a variable HPS fibrinogen concentration (A, B, C, D) and release time (2–24 h). Overlaid lines show average values for a given HPS fibrinogen concentration (*n* = 5). (**F**) Plot showing individual subject analysis for the above conditions. For better overview, lines of average values for each subject were inserted, * = *p* < 0.05 subject 1 vs. 3 (vertical brackets) and condition C vs. D subject 1 (horizontal bracket) (*n* = 3). For C–F the volume of fibrinogen/thrombin added to HPS varied between 0.1–0.2 mL, as shown (A, B, C, D). Error bars represent s.d.

Quantification of the amount of angiogenic factor proteins retained within HPS–fibrin hydrogels, following 24 h release on a low-speed rocking platform, was carried out through indirect calculation by subtracting the amount of factor measured in releasates from that originally present in HPS (note: the volume of fluid lost from hydrogels during processing was negligible). A series of increasing releasate volumes (1–3 mL) was tested here as a means of excluding steady state equilibrium conditions between hydrogel and medium (which might theoretically limit factor release from the hydrogel). This analysis showed that both VEGF and TSP-1 were retained within fibrin hydrogels, while importantly, the factor amount retained was found to be independent of the volume of releasate used (i.e., remained constant across the tested releasate volume range), in all subjects tested, suggesting active retention of factors by the hydrogels (Figure 6A). For releasate volumes 1 and 2 mL, the average retention ratio (RR = fraction of HPS factors retained within HPS–fibrin hydrogels) for VEGF (~0.4) appeared greater than that of TPS-1 (~0.2), which correlated with an inverse relation between the two factors with regards to the dilution ratio (DR = dilution of factor concentration in the releasate relative to its HPS concentration) (Figure 6B), suggesting somewhat greater VEGF retention. These differences were, however, not significant, likely due to the significant inter-subject variation in factor levels (Figure 6A). 

Temporal analysis of the retention of HPS angiogenic factor proteins within HPS–fibrin hydrogels was carried out by varying the fibrin hydrogel mass, through changes in HPS fibrinogen concentration (see Figure 5C), and the factor retention time (corresponding to gelling time), before measuring factor concentration in releasates obtained through hydrogel centrifugation in fresh medium. Although VEGF releasate concentration was found to rise proportionally with increasing HPS fibrinogen concentration (9 vs. 18 mg/mL), up to 60 min retention, suggesting greater factor retention with increasing fibrin hydrogel mass, differences were not statistically significant, for all retention times tested (15–240 min), likely due to the large inter-subject variation (Figure 6C). TPS-1 releasate concentration, on the other hand, showed a significant proportional increase between the low and high HPS fibrinogen concentration tested, up to 60 min retention (*p* < 0.05) (Figure 6C). Accordingly, individual subject analysis did reveal significant inter-subject differences in VEGF, but not TSP-1, releasate levels up to 240 min retention, both at the low and high HPS fibrinogen concentration tested (*p* < 0.05) (Figure 6D), indicating that individual differences in initial HPS pro-angiogenic factor concentration could be carried over to factor retention, at both hydrogel mass levels.

To analyze factor release kinetics, HPS–fibrin hydrogels of varying masses were prepared by varying the HPS fibrinogen concentration (9 vs. 18 mg/mL), and incubated in fresh medium for a variable release time (2–24 h) on a low-speed rocking platform. VEGF cumulative releasate levels rose in the first 4 h, before peaking at 8 h and reaching a plateau by 12 h (Figure 6E). Inter-subject significant differences were observed, up to 4 h, only at the high HPS fibrinogen concentration tested (*p* < 0.05) (Figure 6F), suggesting on one hand that time was indeed significant with respect to VEGF release, and on the other, that the greater VEGF release, observed here, originated from initially greater VEGF loading onto high mass hydrogels (under a certain HPS VEGF level). TSP-1 appeared to follow similar release kinetics, with releasate cumulative concentration peaking at 8 h (Figure 6E), although here inter-subject differences were only significant at the low HPS fibrinogen concentration tested (*p* < 0.05) (Figure 6F), implying that the greater TSP-1 release seen was a direct result of the limited factor retention capacity of low mass hydrogels (under a certain level of TPS-1 loading).

### 2.7. Analysis of HPS Concentration-Dependent Induction of Cellular Angiogenic Responses

**Figure 7:**
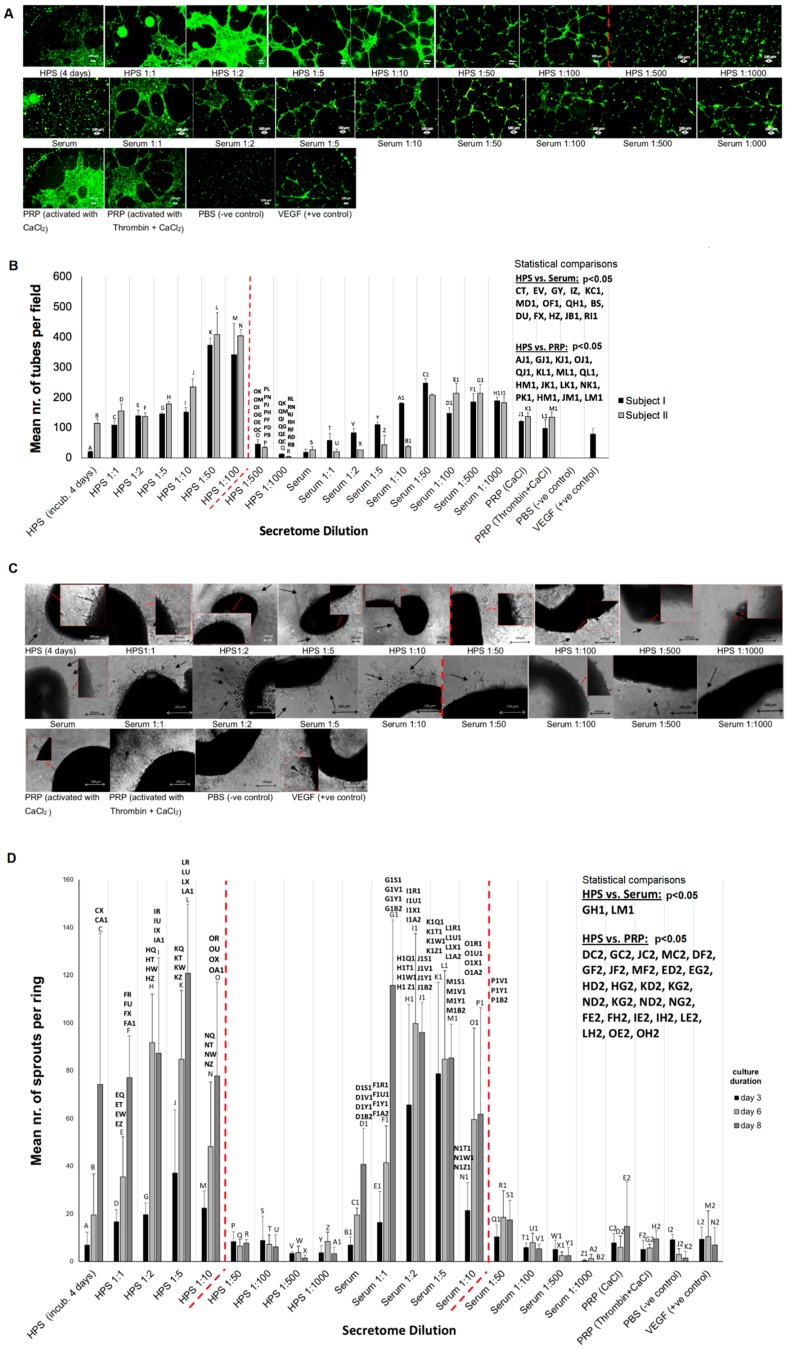
Dependence of in vitro cellular angiogenic responses on HPS growth factor dose. (**A**) Image panel showing the angiogenic response of human umbilical vein endothelial cells, cultured for 12 h under a series of HPS and basal serum dilutions (1:1 to 1:1000), as well as platelet-rich plasma (PRP) activated with thrombin and CaCl_2_ or CaCl_2_ only. (**B**) Plot comparing the induced tube formation for the above conditions (secretomes derived from 2 subjects) (*n* = 3). (**C**) Image panel showing the microvessel sprouting response, tested in the aortic ring model, to a series of HPS and basal serum dilutions (1:1 to 1:1000), as well as PRP activated with thrombin and CaCl_2_ or CaCl_2_ only. Box insets, indicated by red arrows, show enlarged image sections. (**D**) Plot comparing microvessel sprouting for the above conditions under 3, 6, and 8 days of culture (*n* = 3). PBS and VEGF were tested as negative and positive controls, respectively. Statistical comparisons are marked as bold capital letter pairs, corresponding to the data points being compared. Letter pairs shown directly above histograms indicate intra-condition comparisons, while HPS vs. serum and HPS vs. PRP statistical comparisons are shown on the upper right of plot B and D. For all pair comparisons, *p* < 0.05. Red interrupted lines indicate statistically significant cut-off points. Error bars represent s.d.

Release of factors from the fibrin matrix into the surrounding microenvironment evidently results in some degree of reduction in the concentration of factors remaining available within the hydrogel, as well as a dilution of factors in the releasate, relative to their initial HPS concentration (see Figure 6B). In order to define an operational HPS concentration range, we tested the ability of a series of HPS dilutions (1:1 to 1:1000) to induce tube formation and vessel sprouting in vitro. As shown in Figure 7A,B, HPS had an increasingly stronger ability than basal serum for inducing tube formation in Matrigel-seeded endothelial cells, up to 1:100 dilution (*p* < 0.05), where a cut-off was observed; beyond this point (1:500–1:1000 dilution), very little angiogenic response was seen. Interestingly, while a similar initial increase in tube formation was observed in basal serum dilutions, no sharp cut-off was observed here, likely due to the lower initial increase seen compared to the HPS dilutions (Figure 7A,B). Importantly, HPS dilutions of 1:5 to 1:100 were found to induce a significantly stronger angiogenic response than platelet-rich plasma (PRP), regardless of the mode of PRP activation (*p* < 0.05) (Figure 7A,B).

Microvessel sprouting, examined using the aortic ring assay, also demonstrated a HPS factor dose-dependent response, with increasing dilutions correlating with greater sprouting, although here the cut-off point was seen earlier at 1:10 dilution, beyond which sprouting decreased sharply (Figure 7C,D). In contrast to the tube formation assay results, there was little difference between HPS and basal serum dilutions, and also a cut-off point was observed in basal serum cultures, with a rapid decrease in response seen in dilutions greater than 1:10 (Figure 7C,D). Nonetheless, HPS dilutions of 1:1 to 1:10 were found again to be more potent than PRP (regardless of the mode of PRP activation) in inducing vessel sprouting (*p* < 0.05), for all culture periods tested (3, 6, 8 days), in agreement with the tube formation data (Figure 7C,D).

## 3. Discussion

Injectable biomaterial products have become an integral part of modern tissue regeneration strategies [25,26,27,28,29]. However, because most biomaterials only function as passive scaffolds that do not directly activate cellular regeneration processes and tissue remodeling, bio-resorption eventually leads to loss of effect within months [30,31,32]. Furthermore, many materials used are of synthetic or allogeneic/xenogeneic origin, which may produce complications such as immunological reactions or allergies, as well as foreign body granulomas, in some patients [26,30,33]. In contrast, the therapeutic approach presented here is purely autologous and physiological, which, as our preliminary results show (Figure 2), makes it safer for in vivo application. By harnessing the bioactivity of hypoxia preconditioned secretomes, a long-term regenerative effect can be achieved, through cellular remodeling of the HPS–fibrin matrix into native (i.e., collagen-based) extracellular matrix, that complements the early soft tissue support achieved through the interstitial integration of the fibrin hydrogel (Figure 2C). This matrix remodeling may be mediated through the pro-angiogenic effect of HPS on endothelial cells (Figure 4A,B), but also as shown here, via the ability to support fibroblast proliferation and migration toward the target area (Figure 4D,E), processes that are known to be stimulated by FGF, which is present in PBC-derived secretomes [17,18]. From a methodological standpoint, our clinical protocol employs a two-step injection process, i.e., first injection of the HPS–fibrinogen solution, followed by injection of a thrombin–calcium solution for activation (Figure 1B). This allows a minimally-invasive approach, where HPS can be locally injected as a low-viscosity solution, e.g., through micrometer-lumen cannulas, that is homogeneously distributed in the target site, before its activation and resulting gel-matrix formation, which leads to spatial consolidation of HPS protein factors. This effectively makes it possible to precisely position intradermally/subcutaneously a robust bioactive fibrin scaffold, that can persist long enough for tissue remodeling to take place, without the need for surgical implantation. The fact that the HPS–fibrinogen solution is activated and transformed to a gel-matrix *after* injection is also useful for preventing unintended formation of microembolisms, a common complication of direct intravascular injection of gel-based biomaterials (e.g., hyaluronic acid) [34,35]. 

The main aim of this study has been the assessment of the utility of fibrin-based hydrogels as carriers of hypoxia preconditioned PBC-derived growth factors. We had previously found that, on a total protein concentration basis, the releasates of fibrin matrices that were loaded with PBC-derived protein factors contained a significantly greater amount of fibrin(ogen) peptides compared to most other blood-derived peptides detected [4]. Our current findings confirm, however, that these hydrogels can actively retain a range of protein factors released in PBC hypoxic culture media, including pro- and anti-angiogenic factors, but also matrix-remodeling proteins and inflammatory cytokines, some of which (e.g., MMP-9, IL-8) are also pro-angiogenic [18,36] (Figure 3A). It is important to note that despite the presence of inflammatory cytokines, no adverse inflammatory response has ever been recorded in a subject following HPS topical or injectable administration. It might indeed be possible that 4 days of blood incubation, consistently tested here, represents an ideal time-point since this is when the inflammatory phase of wound healing naturally begins to taper, after peaking in the first 24–48 h. Therefore, the concentration of inflammatory cytokines might undergo a gradual reduction as the length of blood incubation increases, owing to a greater rate of protein degradation compared to new protein production. This hypothesis deserves further assessment, within the context of a broader safety study.

From the plethora of pro- and anti-angiogenic factors that we have previously identified in hypoxia pre-conditioned blood-derived secretomes [4,15], as well as those detected here, we have deliberately chosen to examine in more detail two key antagonists, namely VEGF and TSP-1, since these are factors that are known to actively interact with the fibrin matrix [4,23,24]. The statistically significant increase in HPS VEGF concentration with longer blood incubation time, beginning at 48 h and continuing up to 8 days (Figure 3B), confirms our previous findings of hypoxia-induced pro-angiogenic factor upregulation in PBCs [15]. Importantly, the inter-individual variability observed in HPS–fibrin hydrogel and releasate VEGF levels (Figure 3E and Figure 6A), that evidently resulted from inter-subject differences in HPS VEFG concentration (Figure 3C), establishes a basis for the utility of HPS as an autologous, rather than a (pre-fabricated) allogeneic therapy, although the latter approach could also be useful in certain clinical settings, such as acute trauma where there is limited time for a pre-incubation step (note: factor concentration homogeneity in allogeneic compositions could be improved by combining HPS from a wide pool of different subjects). In contrast to VEGF, the anti-angiogenic factor TSP-1 showed no significant variation with the length of the blood incubation period, despite a clear trend for early upregulation and later downregulation (Figure 3B), which has previously been suggested to be a hypoxia-driven phenomenon that promotes the switch towards an angiogenic phenotype [15,37]. The lack of statistical significance seen here could be due to the increased baseline TPS-1 serum level, resulting from platelet activation and TSP-1 release [38], and/or the large statistical variation obtained between subjects. 

With regards to freeze storage of HPS–fibrin hydrogel releasates, we found a significant decrease in VEGF, but not TSP-1 concentration following 4 weeks of storage at −20 °C (Figure 3F). This apparent relative difference in factor degradation was likely due to the initially smaller amount of VEGF in HPS releasates, which makes any decrease appear more significant since the remaining amount is less detectable, rather than a true difference in protein sensitivity to low temperature (although this cannot be excluded through this data). Nonetheless, freeze-stored releasates derived from 4-day incubated HPS were found to contain significantly more VEGF and TSP-1 than freeze-stored basal serum-derived releasates (no blood incubation), indicating that the hypoxia-induced differences in factor concentration persisted following freezing. Importantly, frozen HPS samples were found to maintain their ability to induce tube formation in vitro, although more endothelial cell clustering was observed here compared to fresh HPS samples (Figure 4A). This finding indicates that development of a native-like, and by extension, functional vascular architecture critically depends on the overall balance of pro- and anti-angiogenic protein factors, which was indeed found to be disturbed through freezing, e.g., the VEGF-to-TSP-1 ratio was reduced for 2 day blood incubation samples, rather than simply on the presence of pro-angiogenic factors. Despite this possible reduction in bioactivity, frozen HPS mixtures could be useful for on-demand (i.e., off-the-shelf) use, when there is chronic pathology (e.g., diabetic wound ulcers), and therefore need for repeated secretome administration. This could significantly reduce the number of blood collection sessions required, by providing a large growth factor reserve for continuous application. Freeze storage could further be useful for stocking and transporting allogeneic compositions, making them widely available. 

We could show that the mass of HPP–fibrin hydrogels comprising no exogenous fibrinogen was independent of the blood incubation time (Figure 5A), indicating little or no degradation of endogenous fibrinogen in plasma over 4 days in vitro. A significant increase in HPP– and HPS–fibrin hydrogel mass was, however, observed after increasing the volume of exogenous fibrinogen added, i.e., the HPP–/HPS–fibrinogen concentration (Figure 5B,C). Our data on HPS–fibrin hydrogel mass indicated efficient (i.e., ≥50% of HPS wet volume) absorption and integration of HPS into the fibrin gel matrix that had formed following thrombin activation, for both HPS fibrinogen concentrations tested (9 and 18 mg/mL) (Figure 5C), which is important when considering clinical application. The mass of HPS–fibrin hydrogels was also greater than that of HPP–fibrin hydrogels, for all corresponding fibrinogen concentrations (Figure 5B,C), indicating a relatively weaker consolidation of HPP–fibrin hydrogels. We postulate that Ca^2+^ chelation by EDTA in HPP could have interfered with thrombin activation. Importantly, HPS–fibrin hydrogel mass was largely independent of gelling time, for all fibrinogen concentrations tested (Figure 5C), indicating rapid and complete gel-matrix formation, following thrombin activation, by 15 min. Fast gelling at physiological temperature (37 °C) is paramount for clinical utility as it ensures limited factor spread/leakage into surrounding tissue following injection. The in vivo diffusion distance of the HPS–fibrinogen solution will essentially depend on the viscosity of the solution, therefore primarily on its fibrinogen concentration, but other factors such as HPS dose/volume, rate of injection, needle size, number of injections, target site selection, the presence of tissue damage at the injection site, etc., might also affect diffusion. Further studies could focus on clarifying the influence of these factors. Photographs and 3D scans of hydrogels also demonstrated that larger hydrogel volumes can be obtained with increasing HPS fibrinogen concentration (Figure 5D–F), although the volumetric increase measured was not statistically significant, possibly due to the low sensitivity of the scanning process in association with the limited number of samples tested (note: fluid loss during gel processing/scanning could also have been a contributing factor). Nonetheless, the fact that hydrogel mass was directly proportional to the volume of exogenous fibrinogen added to HPS, indicates that the degree of true, mass-based soft tissue support (i.e., not merely free-fluid or oedema) achieved in the early stage of treatment could be controlled through this method. More importantly, adjustment of the fibrin matrix mass through control of HPS fibrinogen concentration could ensure that matrix integrity is, at least partially, maintained until cellular infiltration and natural remodeling takes place, i.e., the matrix is not completely resorbed through fibrinolysis before this occurs. 

Fibrin-based hydrogels were found to retain angiogenesis-related HPS growth factors, with VEGF appearing to be slightly more retained than TSP-1 (Figure 6A,B). Additionally, there were significant inter-subject differences in the amount of either factor retained, mirroring our data on inter-subject variability in factor releasate levels (Figure 3E). Fibrin has been previously shown to bind VEGF [4,23,39], as well as TSP-1 [4,40]. Indeed, the lack of difference observed in the (calculated) amount of either factor remaining within the fibrin hydrogel, when increasing releasate volumes (1–3 mL) were tested (under max. release time, Figure 6A), suggests that the observed factor retention was not merely the result of a passive factor concentration equilibrium between hydrogel and surrounding medium, indirectly confirming that both VEGF and TSP-1 were actively bound/retained within fibrin matrices. This, combined with the fact that HPS of increasing blood incubation time has a higher VEGF concentration (Figure 3B), further suggests that the fibrin hydrogel has the capacity to maintain the hypoxia-induced differences in pro-angiogenic factor levels generated between basal serum and HPS, through active factor binding.

The data presented here indicate that HPS fibrinogen concentration, and therefore fibrin matrix mass and/or density (which consequently affects the number of factor binding sites and/or the matrix porosity/permeability, as previously proposed [4,39,41,42]) are important parameters for factor retention, although this may become more pronounced as the amount of factor to be retained increases (here, TSP-1 > VEGF, Figure 6C). Importantly, we also found that 15 min of VEGF loading was as efficient as 30–240 min, regardless of the HPS fibrinogen concentration used (Figure 6C), suggesting that even the low hydrogel matrix mass tested was sufficient for rapid VEGF retention. Indeed, inter-subject differences in VEGF retention could be seen at the low, as well as the high HPS fibrinogen concentration (Figure 6D), confirming the ability of low mass hydrogels to also efficiently retain a range of HPS VEGF levels. In comparison, the high HPS fibrinogen concentration correlated with a significantly greater TSP-1 releasate concentration, up to 60 min retention, indicating that a limit in the rate of TSP-1 loading was initially reached at the low hydrogel matrix mass (Figure 6C). As mentioned above, this difference was likely due to the significantly greater amount of TSP-1 than VEGF in HPS. Rapid loading of factors onto the hydrogel, upon its activation/formation in vivo after injection, is a requirement for safe clinical application, since the degree of free factor leakage into surrounding tissue and unwanted side-effects such as oedema/ectopic angiogenesis can be minimized.

**Figure 8:**
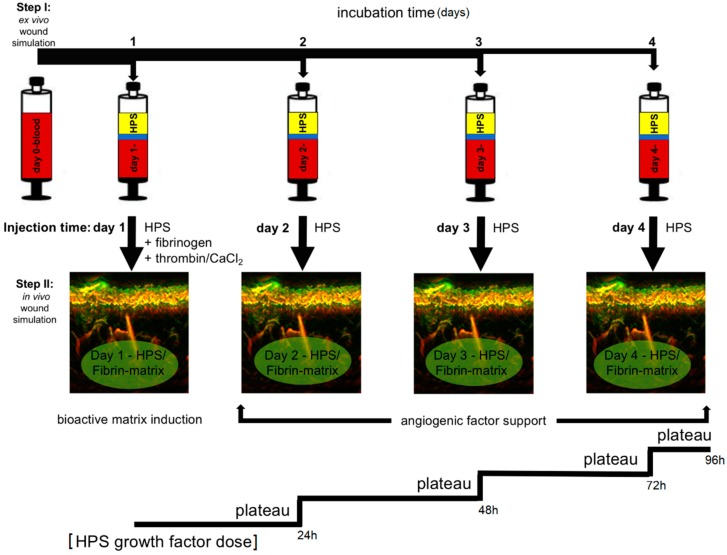
Schematic representation of an injectable therapeutic strategy with temporally-defined HPS fractions. HPS is obtained through the process of extracorporeal wound simulation (see Figure 1), with a variable blood incubation period of 1 to 4 days. Through this process, multiple compositions can be obtained at defined intervals, that comprise different growth factor concentrations and ratios, since hypoxia-induced factor expression by PBCs varies over time. Each composition can be injected at corresponding time intervals (i.e., every 24 h) in order to recapitulate the tissue regeneration-promoting sequence that physiologically occurs after wounding. The combination of HPS with fibrinogen/thrombin at the first injection time point provides an initial bioactive HPS–fibrin matrix scaffold, which can then be vascularized and remodeled into native tissue through gradual growth factor release. Continuous HPS-derived angiogenic factor support, at predefined release-plateau time points, ensures a steady increase in HPS growth factor dose, and a sustainable cellular angiogenic response. Note, however, that this pattern of administration is not mandatory, i.e., wider time intervals may be used between injections, according to the clinical setting.

Analysis of the rate of factor release from HPS–fibrin hydrogels showed that both VEGF and TSP-1 were released as early as 2 h, regardless of fibrin hydrogel matrix mass, reaching a plateau at 8–12 h (Figure 6E). This plateau could be the result of a passive factor concentration equilibrium between hydrogel and surrounding medium, but also as previously discussed, active factor retention by the hydrogel, which ensures that once loosely-trapped factors have been eluted, release then slows down. This suggests, on one hand, that a proportion of (bound) HPS factors would remain available for cells that migrate into the matrix (i.e., the fibrin matrix remains bioactive following initial factor release, and therefore can be optimally remodeled/vascularized), and on the other, that repeated HPS administration might be necessary to promote and maintain a robust angiogenic response in vivo, since growth factor proteins generally have a short half-life, e.g., VEGF ~60 min [43] (see last paragraph and Figure 8). As expected, the influence of fibrin hydrogel mass on factor retention also extended to factor release, reflected by inter-subject differences in VEGF and TSP-1 releasate levels, with the higher matrix mass correlating with more efficient loading, and subsequently greater release, for low concentration (and/or low molecular weight) factors (e.g., VEGF), and the lower matrix mass correlating with limited factor retention capacity, and subsequently greater release, for high concentration (and/or high molecular weight) factors (e.g., TSP-1) (Figure 6F). This is indeed not surprising, since matrix mass effectively determines the number of factor-binding sites, but it essentially underscores the key role active factor retention might play as the main regulator of factor release, which consequently may become a secondary (i.e., passive) phenomenon in vivo, dependent on fibrin matrix degradation, i.e., fibrinolysis, and cellular remodeling. It might therefore be possible to indirectly control the rate of factor release from fibrin hydrogels, through adjustment of the HPS fibrinogen concentration, i.e., the matrix mass/density. Controlled release is a prerequisite for establishing stable chemotactic gradients that directionally drive cellular migration towards the matrix, thus enabling its function as factor carrier to facilitate its utility as cellular scaffold [44]. Previous studies have demonstrated that in vivo implanted fibrin matrices containing growth factors (e.g., VEGF) can induce local growth of blood vessels [45,46,47,48,49], while matrix vascularization and integration into the recipient tissue is accompanied by the migration and proliferation of various cell types, e.g., fibroblasts, smooth muscle cells, keratinocytes [41,48]. Work from this group has also shown that the carrier property of the fibrin matrix enables it, in a mass-dependent manner, to seamlessly perform a dual role during wound healing, initially acting as an anti-angiogenic haemostatic barrier, and later as angiogenic scaffold, through changes in the balance of releasate pro- and anti-angiogenic factor concentrations [4]. Therefore, HPS growth factor delivery in vivo through a tunable fibrin matrix carrier is a rational approach, since it practically utilizes the same vehicle as the one normally employed during native tissue repair [24,41,42].

Gradual release of growth factors from the fibrin hydrogel into the surrounding microenvironment inevitably leads to a reduction in the concentration of factors remaining available to cells that migrate into the matrix, but also a dilution of factors in the releasate with respect to their initial HPS concentration (Figure 6B), the latter being dependent on the degree of factor-specific retention by the matrix and the factor diffusion distance. In contrast to being prohibitive to the secretome’s functionality, our data clearly show that HPS dilution, up to an extent, leads to an improved angiogenic response in terms of vessel sprouting (up to 1:10 dilution) and tubulogenesis (up to 1:100 dilution) (Figure 7). Additionally, our ability to control the fibrin matrix mass, by adjusting the HPS fibrinogen concentration, and therefore the rate of factor retention and consequently release, could provide a useful tool for fine-tuning the induced cellular responses. Future work could focus on analyzing the spatio-temporal concentration profiles of individual growth factors within the matrix-surrounding milieu, which could allow predictions to be made about complex cellular angiogenic patterns in vivo. With regards to the mechanism(s) that might be responsible for the bell-shape profile of the angiogenic responses observed (Figure 7B,D), we propose that this might be mediated through changes in the balance, i.e., *relative* concentrations of pro- and anti-angiogenic factors present in HPS, such as VEGF and TPS-1. Logically then, the optimal dilution range for the most favorable response is such that allows a relative reduction of angiogenesis-inhibitory factors, while maintaining an adequate amount of pro-angiogenic factors. Predictably, when the latter becomes too low, the response sharply decreases, as shown in our data. This mechanism would be in support of angiogenic *dis*inhibition, instead of direct pro-angiogenic stimulation, as the primary switch for triggering hypoxia-induced angiogenesis, as previously hypothesized [4]. This hypothesis is indeed supported by our finding that HPS of certain dilutions could induce a stronger angiogenic response than PRP (Figure 7), which is by its nature a concentration product, and is known to contain large amounts of angiogenesis-inhibiting (platelet-derived) growth factors, e.g., PF4, TSP-1 [50]. Further work is nonetheless required before the relative differences in factor profile between these secretomes, that are responsible for such effects, can be more precisely defined.

HPS–fibrin hydrogels present an opportunity for a paradigm shift in the utilization of injectable compositions, away from that of inert biomaterials that primarily induce a foreign body reaction, toward biomimetic and bioactive scaffolds. This approach is truly aligned with the understanding that tissue regeneration and new tissue formation can only occur within a physiological structural foundation, analogous to that present following wounding. HPS–fibrin hydrogels comprise the correct biochemical signaling, i.e., hypoxia-induced growth factors, and biomaterial properties, i.e., fibrin-based growth factor carrier, required for this purpose. Accordingly, HPS–fibrin hydrogels could offer an improved alternative to platelet-rich products (e.g., PRP/platelet-rich fibrin matrix (PRFM)) by providing more optimized and temporally-defined angiogenic compositions, based on PBCs’ natural and timely variable responses to hypoxic stress, since hypoxia-induced angiogenic factor expression varies significantly over time (see Figure 3B), rather than merely the static collection of factors already present within platelets at the time of blood collection [15,22,51]. This would allow the development of therapeutic strategies that employ controlled HPS growth factor dosage, resulting in a more sustainable in vivo response (Figure 8), and therefore merits further animal-based and clinical investigation. The cell-free nature of these compositions also makes them safe and suitable for off-the-shelf use via freeze-storage.

## 4. Materials and Methods

### 4.1. In Vitro Skin Irritation Test of HPS Topical Administration in the EPISKIN Model

EpiSkin^TM^ test of a HPS-containing cream was performed to predict its irritation potential by measurement of its cytotoxic effect, as reflected in the MTT assay, according to the Organisation for Economic Co-operation and Development (OECD) Test Guideline No. 439, 26 July 2013 (Study number: 792.554.4509 and 792.554.4602). HPS was obtained following 4 days of blood incubation (for more details see Section 4.4) and mixed into a commercial cream base (dermatologically tested to be safe for topical application) at a concentration of 1 or 5%. Disks of EPISKIN (three units/chemical) were treated with test material and incubated for 15 min at room temperature. Exposure of test material was terminated by rinsing with PBS 1× solution. Epidermis units were then incubated at 37 °C for 42 h in an incubator with 5% CO_2_. The viability of each disk was assessed by incubating the tissues for 3 h with MTT solution at 37 °C in 5% CO_2_ protected from light. The precipitated formazan was then extracted using acidified isopropanol and quantified spectrophotometrically. Sodium Dodecyl Sulphate (SDS) 5% aq.solution and PBS-treated epidermis were used as positive and negative controls, respectively. For each treated tissue, viability was expressed as a percentage relative to negative control. The test material was considered to be an irritant to skin, if the mean relative viability after 15 min of exposure and 42 h post incubation was ≤50% of the negative control. Positive and negative controls showed the expected cell viability values within acceptable limits. Three samples were tested per condition.

### 4.2. In Vivo Topical Administration of HPS in a Chronic Wound Ulcer

A 63-year-old female patient presenting in our clinic (Department of Experimental Plastic Surgery, Clinic for Plastic, Reconstructive and Hand Surgery, Klinikum rechts der Isar, Technische Universität München, Munich, Germany) with a chronic venous leg (pretibial) ulcer (~3 cm diameter) underwent gold-standard treatment with repeated wound debridement and split-thickness skin grafting (STSG). Following a failed attempt to treat the ulcer for 7 months, a ‘last-resort’ treatment with a 5% HPS-containing ointment (HPS obtained following 4 days of blood incubation) was undertaken. The subject provided written informed consent before treatment. The ulcer was followed during the treatment period (3 months) with clinical photography and measurement of its base diameter.

### 4.3. Analysis of Local Tissue Distortion by HPS–Fibrin Hydrogel through Ultra-Broadband Raster-Scanning Optoacoustic Mesoscopy Technique (UB-RSOM)

A healthy subject (male, age 25) receiving a dermal treatment with HPS–fibrin hydrogel, first received a forearm injection for compatibility testing, as per our clinic’s (Klinikum rechts der Isar, Technical University Munich, Munich, Germany) standard protocol. The subject provided written informed consent before treatment. In the first step, 1 mL HPS (obtained following 4 days of blood incubation) combined with 0.2 mL fibrinogen solution (human fibrinogen 90 mg/mL, aprotinin 3000 KIU/mL, factor XIII 10–50 IU/mL, fibronectin 2–9 mg/mL; Baxter, Unterschleißheim, Germany) was injected in the forearm (20 G needle, B Braun AG, Melsungen, Germany), in the superficial dermis. In the second step, 0.2 mL of thrombin (500 IU/mL)/calcium chloride (40 µmol/mL) solution (Baxter, Germany) was injected at the same site, for HPS activation (Figure 1B) (note: due to the high viscosity the fibrin gel has once it is formed, which makes injection difficult, the thrombin solution is injected subsequently to the injection of the HPS/fibrinogen solution, in order to facilitate uniform and pain-free injection). The injection site was chosen in close proximity to a nevus at the forearm, as a reference point for image registration and correlation. To assess the distortion effect caused in the dermis by the HPS injection an ultra-broadband hand-held clinical raster scan optoacoustic system (UB-RSOM) was used [52]. The UB-RSOM system relies on broadband detection of optoacoustic waves generated by label-free biomolecules in skin exposed to pulsed laser light [53]. The ultrasound waves were collected in raster scanner mode from the region of interest by a customized spherically focused piezoelectric transducer (Sonaxis SA). To ensure the safety of the experiment the irradiance delivered to the skin surface did not exceed 3.75 µJ/mm^2^ at a 500 Hz repetition rate [54]. Cross-sectional views of human forearm skin were obtained by calculating maximum intensity projection of 3D reconstructed optoacoustic images. For representation purposes, and to underline small features, optoacoustic signals were filtered in two bandwidths: 14–40 MHz (low frequencies, big objects) and 40–120 MHz (high frequencies, small objects). The displayed images were color-coded in red and green colors, representing low and high frequencies, respectively [55]. The HPS gel substance was transparent for the utilized technique and did not generate optoacoustic signal. We performed acquisitions on day 0, 1, 3, 5, and 7 following HPS injection (Day 0 acquisition was taken as a reference before injection). The imaging site slightly varied in inter-day manner, due to positioning of the UB-RSOM interface onto the skin surface.

### 4.4. Preparation of Hypoxia Preconditioned Serum/Plasma (HPS/HPP) and HPS/HPP–Fibrin Hydrogels

All blood donors provided written informed consent as directed by the ethics committee of the Technical University Munich, Germany, which approved this study (File Nr.: 498/16S). When blood was taken several times from the same subject, care was taken to avoid significant lifestyle changes (e.g., nutrition, alcohol, smoking). In the first step, 10 mL peripheral venous blood was collected in a 10 mL polypropylene syringe (Omnifix^®^, B Braun AG, Germany) for HPS preparation or in blood tubes filled with EDTA anticoagulant (BD Vacutainer, Becton, Dickinson and Company, USA) for HPP preparation. Five milliliters of air was then drawn into the syringe through a 0.2 µm filter (Sterifix^®^, B Braun AG, Germany), with the plunger fully withdrawn. The EDTA tube was opened to allow air to enter and re-sealed with foil. Subsequently, the syringe and the EDTA tube were placed upright in the incubator (37 °C/5% CO_2_) and incubated for 2 to 8 days (blood incubation time). Pericellular (local) hypoxia (~1% O_2_) was induced in situ through cell-mediated O_2_ consumption, by controlling the blood volume per unit area (BVUA ≥ 1 mL/cm^2^), and consequently the PBC seeding density in the blood container, as previously defined through white blood cell count [4,21]. After incubation, the blood was separated through sedimentation into three layers (from top to bottom: serum/plasma, clot/buffy coat, red blood cell component), so that the top layer (HPS/HPP) could be filtered (Sterifix^®^, B Braun AG, Germany) in a new syringe, removing cells/cellular debris. Fibrin hydrogels of varying mass were formed by combining varying volumes (0.1 or 0.2 mL) of fibrinogen solution (human fibrinogen 90 mg/mL, aprotinin 3000 KIU/mL, factor XIII 10–50 IU/mL, fibronectin 2–9 mg/mL; Baxter, Germany) with 1 mL of HPS or HPP. After mixing, the solution was combined with a varying volume (0.1 or 0.2 mL) of thrombin (500 IU/mL)/calcium chloride (40 µmol/mL) solution (Baxter, Germany) for activation. Following completion of a variable gelling time (15 min, 30 min, 1 h, 2 h, 4 h) in the incubator (37 °C), HPS–/HPP–fibrin hydrogels were transferred to a 15 mL tube, and 1 mL phosphate buffered saline (PBS) fresh medium was added, before gels were centrifuged (2000 RPM, 4 °C, 15 min) or fixed on a low-speed rocking platform for a varying release time (2 h, 4 h, 8 h, 12 h, 24 h) in order to obtain releasates. Releasates for each condition were then sampled and analyzed.

### 4.5. Proteome Assay of Fibrin Matrix Releasates

Fibrin matrices were cultured in the hemostatic components model (HCM), as previously described [4]. Briefly, the buffy coat was isolated from 10 mL peripheral blood, by centrifugation in EDTA-Vacutainer tubes (BD, Germany) at 3000 rpm/4 °C for 15 min, and reconstituted in 10 mL serum-free (SF) medium (AIM V, Invitrogen, Bremen, Germany). One milliliter of blood cell/SF medium mixture was added to type I collagen-coated wells (area ~10 cm^2^) containing 2 mL SF medium. Fibrin matrices were formed by combining 1 mL fibrinogen solution (fibrinogen 90 mg/mL, aprotinin 3000 KIU/mL, factor XIII 10–50 IU/mL, fibronectin 2–9 mg/mL) (Baxter, Germany) with 1 mL thrombin (500 IU/mL)/calcium chloride (40 µmol/mL) solution (Baxter, Germany), in cell-culture inserts with a 1μm pore polyethylene terephthalate (PET) membrane (BD, Germany), before adding 1 mL SF medium. Inserts were then transferred into the PBC-containing collagen-coated wells. Culture was carried out under hypoxia (3% O_2_) within a 37 °C/5% CO_2_ incubator. Following 7 days of culture, fibrin matrices were removed from inserts and added to 1 mL fresh SF medium (albumin-free medium was used to reduce interference with peptide detection), then centrifuged at 3000 rpm for 15 min, to obtain cell-free releasates. Fibrin matrix releasates were analyzed with Angiogenesis Proteome Profiler array (RnD Systems, inc., Minneapolis, USA), according to manufacturer’s instructions. SF medium was tested as a negative control. Reference spots (positive control) were used to align the transparency overlay template and to confirm that the array had been incubated with Streptavidin-HRP during the assay procedure. Quantification of relative factor levels (sample signal/reference signal) was carried out by image analysis of scanned X-ray film images (3, 4, and 5 min exposures) using an imaging software (Image J, NIH, USA). An averaged background signal was subtracted from the average pixel density of each pair of duplicate spots. Three samples were tested.

### 4.6. Analysis of the Effect of Varying Blood Incubation Length on HPS and HPS–Fibrin Hydrogel Releasate VEGF and TSP-1 Concentration

Peripheral venous blood obtained from five healthy subjects (*n* = 5) was incubated for 0, 2, 4, and 8 days. HPS was analyzed by ELISA for VEGF and TSP-1 according to manufacturer’s (RnD Systems, inc., Minneapolis, USA) instructions. Fibrinogen (0.2 mL) and thrombin/Ca^2+^ (0.2 mL) was then added to 1 mL HPS/HPP to prepare fibrin hydrogels, as described above. Gelling time (corresponding to factor retention time) amounted to 4 h, after which releasates were obtained through centrifugation of gels in 1 mL PBS for 15 min (2000 RPM), and analyzed by ELISA for VEGF and TSP-1 according to manufacturer’s (RnD Systems, inc., Minneapolis, USA) instructions, directly after collection or following 4 weeks freeze storage at −20 °C. Five hydrogels were tested per condition.

### 4.7. Assessment of the Effect of HPS on Endothelial Cell Tube Formation/Vessel Sprouting and Dermal Fibroblast Proliferation/Migration

HPS was prepared as described above (4 and 8 days of blood incubation), and tested directly or after freeze storage at −20 °C for 4 weeks. For angiogenesis experiments, HPS and basal serum were reconstituted with phosphate buffered saline (PBS) to create a dilution series ranging from 1:1 to 1:1000 final concentration. PBS and VEGF were tested as negative and positive controls, respectively, as indicated. AIM V serum-free medium (Thermo Scientific, USA) was used as negative control when no HPS dilution occurred. Platelet rich plasma, prepared as previously described [56] and activated with either thrombin and CaCl_2_ (Tisseel, Baxter, Germany) or by CaCl_2_ only, was included as an additional test condition in certain angiogenesis experiments, as indicated. For fibroblast experiments, HPS and basal serum were reconstituted at a 10% final concentration in a solution of Dulbecco’s Modified Eagle’s Medium (DMEM). For fibroblast proliferation experiments DMEM without or with 10% fetal calf serum (FCS, commonly used for fibroblast culture) was tested as negative and positive controls, respectively. For fibroblast migration experiments, DMEM and TNFα (regulator of fibroblast migration) were tested as negative and positive controls, respectively.

The angiogenic potential of HPS- and serum-containing media was tested in an in vitro angiogenesis assay, by assessing their ability to induce tube formation in human umbilical vein endothelial cells (HUVECs, CellSystems, Germany) seeded on factor-reduced Matrigel (BD, Germany). HUVECS were seeded at a density of 10 × 10^3^/well, with 50 μL of test or control media added per well (μ-Slide Angiogenesis, Ibidi, Germany), and cultured in 5% CO_2_/37 °C for 12 h. Cells were then stained with Calcein AM (PromoKine, Germany) and tube formation was observed with phase contrast microscopy. Assessment of the extent of capillary-like network formation was carried out by counting the number of tubes per field. Secretomes were also tested in the aortic ring model to assess their ability to induce microvessel sprouting. Aortic rings were dissected from female adult mice as previously described [57], underwent overnight serum starvation in opti-MEM reduced serum medium (Life Technologies, Germany) and embedded into Matrigel bilayer matrix (50 μL/layer in 96-well plates) (BD, Germany). Secretomes and control media were added (150 μL/well) to the rings, before culturing them in 5% CO_2_/37 °C. Medium change was carried out every 3 days, while rings were observed with phase contrast microscopy at 0, 3, 6, and 8 days and photographed, with all 4 quarters per ring analyzed for sprouting (formation of structures of connected cells that are attached, at their base, to the ring). Three rings were tested per condition. 

For assessment of cell proliferation, human dermal (foreskin) fibroblasts (Hs27 line) were seeded in wells at a density of 1 × 10^4^ cells/well and cultured in media containing 10% HPS or basal serum for 72 h. Fibroblast proliferation was assessed with the alamar blue assay, which incorporates an oxidation–reduction indicator that both fluoresces and changes color in response to chemical reduction of growth medium resulting from cell growth. The alamar blue assay was performed by adding alamarBlue reagent directly to cells in culture medium in an amount equal to 10% of the volume in the well and incubating cultures with alamarBlue for 4 h at 37 °C in a 5% CO_2_/37 °C cell culture incubator, protected from direct light. Fluorescence was then measured using a fluorescence excitation wavelength of 570 nm. Fibroblast migration was assessed in real-time with the xCELLigence^®^ system (ACEA Biosciences, Inc., USA), using a fibroblast seeding density of 4 × 10^4^ cells/well. The ability of DMEM medium containing 10% HPS or basal serum to act as chemoattractant was tested for 26 h, as cells moved from the upper chamber towards the chemoattractant in the lower chamber through an 8 μm pore membrane. Five samples were tested per subject per condition. 

### 4.8. Analysis of the Effect of Varying HPS/HPP Fibrinogen Concentration on HPS/HPP–Fibrin Hydrogel Mass

HPS– and HPP–fibrin hydrogels were prepared as described above. The incubation time of peripheral venous blood obtained from five subjects (*n* = 5) was set at 4 days, unless otherwise indicated. The volume of fibrinogen and thrombin/Ca^2+^ solution added to HPS/HPP varied from 0.1 to 0.2 mL, as indicated. Gelling time for HPS–fibrin hydrogels was 15 min, 30 min, 1 h, 2 h, and 4 h, as indicated. For the experiments on HPP–fibrin hydrogels, a gelling time of 15 min was selected. After preparation, HPS–/HPP–fibrin hydrogels were weighed and photographed (note: no washing of the gels was performed prior to measurements, in order to prevent fluid loss from the hydrogel during processing). Five hydrogels were tested per condition.

### 4.9. Scan-Assisted Quantification of HPS–Fibrin Hydrogel Volume Differences

HPS–fibrin hydrogels were prepared as described above. The incubation time of peripheral venous blood obtained from four subjects (*n* = 4) was set at 4 days. The volume of added fibrinogen varied from 0.1 to 0.2 mL, as indicated. Gelling time amounted to 4 h. Volumetric analysis was performed based on 3D surface scans of hydrogels. Four hydrogels per condition were scanned with a structured light 3D scanner (Spider, Artec, Luxembourg) under standardized lighting conditions. Light reflections were reduced by polysaccharide coating (StarSil; Hemotec Medical GmbH, REF SS005) of the gels. The scans were recorded and digitally converted into virtual 3D models using Artec Studio 12 (Artec, Luxembourg), while final volume measurements were carried out using specific software (Geomagic Studio, 3D Systems, Rock Hill, SC, USA).

### 4.10. Analysis of the Factor Retention Capacity of HPS–Fibrin Hydrogels

HPS–fibrin hydrogels were prepared as described above. The incubation time of peripheral venous blood obtained from five subjects (*n* = 5) was set at 4 days. Following blood incubation, 1 mL of HPS was collected and 0.2 mL fibrinogen and thrombin/Ca^2+^ were then added to form hydrogels. Factor retention time, corresponding to gelling time, amounted to 4 h. Releasates were obtained by incubating the gels in 1, 2, or 3 mL PBS on a low-speed rocking platform for 24 h at room temperature. Releasates for each condition were then sampled and the exact releasate volume was determined. Care was taken to prevent fluid loss from gels during handling. HPS and releasates were analyzed by ELISA for VEGF and TSP-1 according to manufacturer’s (RnD Systems, inc., Minneapolis, USA) instructions. After first measuring the amount of factor present in HPS and releasates, the amount of factor remaining in the hydrogel was indirectly calculated using the following equation:

HPS _hydrogel_ factor = HPS factor − HPS _releasate_ factor

The factor retention ratio (RR), i.e., the fraction of HPS factors retained in the hydrogel following 24 h release, was calculated using the following equation:

RR = HPS _hydrogel_ factor/HPS factor

Five hydrogels were tested per condition.

### 4.11. Analysis of the Effect of Varying HPS–Fibrin Hydrogel Mass on the Rate of Factor Retention and Release

HPS–fibrin hydrogels were prepared as described above. The incubation time of peripheral venous blood obtained from five subjects (*n* = 5) was set at 4 days. The volume of added fibrinogen and thrombin/Ca2+ varied between 0.1 mL to 0.2 mL, as indicated. For assessing factor retention rate, the factor retention time (corresponding to gelling time) was varied between 15 min, 30 min, 1 h, 2 h, and 4 h, after which releasates were obtained through centrifugation of gels in 1 mL PBS for 15 min (2000 RPM) at room temperature. For assessing factor release rate, the factor retention time was set at 4h, and releasates were obtained by incubating the gels in 1 mL PBS on a low-speed rocking platform for a variable release time of 2 h, 4 h, 8 h, 12 h, and 24 h, at room temperature. Releasates for each condition were then sampled and analyzed by ELISA for VEGF and TSP-1 according to manufacturer’s (RnD Systems, inc., Minneapolis, USA) instructions. Five hydrogels were tested per condition.

### 4.12. Statistical Analysis

For each experimental condition *n* = 5 subjects were tested (in certain cases *n* = 3 or 4 subjects were tested, as noted). Data are expressed as mean ± standard deviation. Statistical analysis was carried out using Student’s independent *t*-test where a maximum of two groups was compared or repeated-measures ANOVA with Bonferroni adjustment, accompanied by post-hoc pairwise comparisons for analysis of three or more groups, using SPSS 14 software. Mauchly’s test was used to assess violation of sphericity in repeated-measures ANOVA, and in instances where Mauchly’s test was significant, degrees of freedom were corrected using Greenhouse–Geisser estimates of sphericity. The probability of a type one error was set to 5% (α = 0.05), unless noted otherwise. 

## 5. Conclusions

This study presents a novel method for controlled delivery of PBC-generated, hypoxia-induced growth factor mixtures in cell-free HPS–fibrin hydrogel carriers. Our data indicate that pro- and anti-angiogenic factor loading and release kinetics can be precisely controlled by adjusting the HPS fibrinogen concentration, and therefore the mass (and volume) of the fibrin hydrogel matrix. This could consequently allow modulation of cellular angiogenic responses in vivo, in a growth factor dose-dependent manner. From both a biochemical and biomaterial standpoint, this approach lays the foundation for developing an autologous, bioactive injectable composition to clinically target tissue repair and regeneration.

## 6. Patents

A device for preparation of hypoxia preconditioned blood-derived secretomes and one-step factor loading onto sterile carriers was filed in 2013 by the Technische Universität München—Klinikum rechts der Isar: “Device-based methods for localised delivery of cell-free carriers with stress-induced cellular factors” WO2013113821A1, WIPO (PCT).

## Abbreviations

PBC	peripheral blood cells
HPS	hypoxia preconditioned serum
HPP	hypoxia preconditioned plasma
PRP	platelet-rich plasma
PRFM	platelet-rich fibrin matrix
VEGF	vascular endothelial growth factor
TSP-1	thrombospondin-1
IL-8	interleukin-8
FGF	fibroblast growth factor
MMP-9	matrix metalloproteinase-9
PF-4	Platelet Factor 4
PBS	phosphate buffered saline
